# Definitive chemoradiotherapy combined with anti-PD-1 immunotherapy for inoperable esophageal squamous cell carcinoma: a multicenter real-world study

**DOI:** 10.1080/15384047.2025.2504726

**Published:** 2025-05-14

**Authors:** Xiongtao Yang, Xiaomin Wang, Qin Xiao, Xiaolin Ge, Nuo Yu, Jiao Li, Guojie Feng, Ziyu Zheng, Yingying Jiang, Lin Lu, Xiaojie Xia, Lei Deng, Tao Zhang, Wenqing Wang, Wenyang Liu, Jianyang Wang, Zefen Xiao, Zongmei Zhou, Nan Bi, Hui Wang, Cheng Chen, Xin Wang

**Affiliations:** a Department of Oncology, Beijing Changping Hospital, Beijing, China; b Department of Radiation Oncology, National Cancer Center/National Clinical Research Center for Cancer/Cancer Hospital, Chinese Academy of Medical Sciences and Peking Union Medical College, Beijing, China; c Department 1st of Radiation Oncology, Anyang Cancer Hospital, Anyang, Henan, China; d Department of Radiation Oncology, Hunan Cancer Hospital and The Affiliated Cancer Hospital of Xiangya School of Medicine, Central South University, Changsha, Hunan, China; e Department of Radiation Oncology, Jiangsu Province Hospital and Nanjing Medical University First Affiliated Hospital, Nanjing, Jiangsu, China; f Department of Oncology, Province Geriatric Hospital, Nanjing, Jiangsu, China; g Department of Radiotherapy, The Affiliated Cancer Hospital of Nanjing Medical University, Nanjing, Jiangsu, China

**Keywords:** Immunotherapy, definitive chemoradiotherapy, inoperable esophageal cancer, real-world study, PD-L1

## Abstract

The potential benefits of adding immune checkpoint inhibitors (ICIs) to definitive chemoradiotherapy (dCRT) for inoperable esophageal squamous cell carcinoma (ESCC) remain unclear, different timing for immunotherapy intervention during the peri-dCRT period on survival outcomes is worth exploring. Patients with inoperable ESCC receiving dCRT combined with ICIs between April 2018 and April 2022 were recruited from five hospitals in China. A historical control group treated with dCRT alone was used for comparison. Stabilized inverse probability of treatment weights (sIPTW) analyses were conducted to compare survival. The survival outcomes and treatment-related adverse effects were analyzed. A total of 290 patients with inoperable ESCC who received dCRT+ICI treatment were analyzed. The median follow-up was 35.7 months. The 1-year and 2-year overall survival (OS) rates were 86.7% and 66.9%, respectively, and the 1-year and 2-year progression-free survival (PFS) rates were 66.7% and 47.3%, respectively. The median PFS was 22.4 months, and the median OS was not reached. The dCRT+ICI group had significantly longer OS than the historical control group (2-year OS: 66.9% vs. 56.5%, for sIPTW-adjusted data: HR = 0.62, *p* < .001). After sIPTW, no differences in OS were found among the induction, concurrent, and consolidation groups. The sIPTW-adjusted analysis showed that both the induction and concurrent groups had significantly longer PFS than the consolidation group. Combining immunotherapy with dCRT improved survival outcomes and demonstrated a favorable safety profile in patients with inoperable ESCC. Induction or concurrent immunotherapy may provide superior survival benefits compared to consolidation immunotherapy.

**Trial registration:**

Trial no. NCT04821778 registered in ClinicalTrials.gov

## Background

Esophageal cancer is one of the most common digestive system tumors. Its main pathological type, squamous cell carcinoma, accounts for about 90% of esophageal cancers in China.^1,2^ The RTOG8501 and RTOG9405 studies have established that definitive chemoradiotherapy (dCRT) is the standard treatment for inoperable locally advanced esophageal squamous cell carcinoma (LA-ESCC), with a 2-year overall survival (OS) of 38–40% and a 5-year OS of 26%.^3–5^ With the advancement of radiation techniques, for example, intensity-modulated radiation therapy, volumetric-modulated arc therapy, and helical tomotherapy, and improvements in economic conditions (such as better nutritional status), the survival rate of LA-ESCC has improved in China. For example, a previous nationwide multicenter real-world study of inoperable ESCC treated with dCRT in China from 2015 to 2016 revealed a 2-year and 5-year OS of 50% and 30%, respectively.^6^ In addition, the results of another prospective, randomized, phase III study on LA-ESCC treatment (ESO-shanghai1, paclitaxel/5-FU vs. cisplatin/5-FU-based dCRT) revealed a 2-year and 5-year OS of up to 60.6–61.5% and 40.8–44.3%, respectively.^7^ However, the overall prognosis still remains poor for patients with inoperable ESCC. Thus, there is a clear need for more effective treatment strategies for these patients.

Several prospective randomized controlled phase III studies have shown that immune checkpoint inhibitors (ICIs) combined with chemotherapy improved the prognosis of patients with advanced esophageal cancer; therefore, ICIs have become the new standardized treatment agents for advanced esophageal cancer.^8–11^ In the case of LA-ESCC, a few prospective single-arm studies with limited sample size have shown that dCRT combined with ICIs (dCRT+ICIs) has controllable safety and satisfactory efficacy (2-year OS: 50.0–75.0%).^12–14^ In particular, based on the results of multiple phase III studies, PD-1 inhibitors combined with chemotherapy have become the standard first-line treatment for advanced esophageal cancer.^8–11^

With regard to the timing of immunotherapy in patients undergoing dCRT+ICI treatment, the PACIFIC study confirmed that dCRT with consolidation immunotherapy (administered after CRT to kill any remaining cancer cells) improved survival in non-small cell lung cancer (NSCLC) compared to dCRT alone. However, the PACIFIC-2 study showed that concurrent immunotherapy did not have positive results.^15^ In contrast, a clinical retrospective study showed that among 758 patients with 1,798 lesions, the concurrent immunoradiotherapy regimen was the most effective. Specifically, concurrent immunoradiotherapy extended OS by approximately 9 months, particularly when immunotherapy was initiated at least 1 month before radiotherapy.^16^ Some studies have shown that induction immunotherapy before radiotherapy can promote vascular normalization and alleviate hypoxia, thereby improving radiosensitivity in locally advanced esophageal cancer.^17^ Thus, the optimal timing for combining immunotherapy with radiotherapy remains undetermined. Currently, most prospective phase III studies are designed around concurrent and consolidation immunotherapy, such as Keynote 975, KUNLUN, and RATIONALE 311.^18–20^ Further, the design of the SKYSCRAPER07 study includes consolidation immunotherapy.^21^ The results of these clinical studies may provide evidence to clinical guide decision making related to the sequence of immunotherapy and radiotherapy.

Based on the current state of and gaps in the research on applying dCRT+ICI treatment in ESCC (discussed above), the aims of this study are (1) to evaluate whether the addition of PD-L1 as an ICI to dCRT could also improve survival outcomes for patients with inoperable ESCC and (2) to explore the impact of different immunotherapy intervention timings on prognosis.

## Materials and methods

### Study design and participants

The data were collected from patients treated with dCRT combined with PD-1 blockade at five hospitals in China between April 2018 and April 2022 (Supplementary table S1). The last follow-up time point was April 2024. Patients who met the following inclusion criteria were included: (1) ESCC confirmed by histological or cytological examination; (2) not suitable for operation due to unresectable disease, surgical contraindications, or refusal to undergo surgical treatment; (3) M1 restricted to supraclavicular lymph node metastases (according to the 8th edition of the American Joint Committee on Cancer); (4) administration of immunotherapy with PD-1 blockade before, concurrently with, or after dCRT; (5) administration of at least one cycle of immunotherapy and chemotherapy during the peri-radiation period; (6) radiation dose for the primary tumor ≥50 Gy; and (7) availability of complete clinical data for retrieval. The exclusion criteria were (1) diagnosis of other malignant tumors, except melanoma, in the recent 5 years; (2) distant metastasis to the non-regional lymph nodes and organ metastasis; and (3) previous chest radiotherapy.

**Figure 1.**
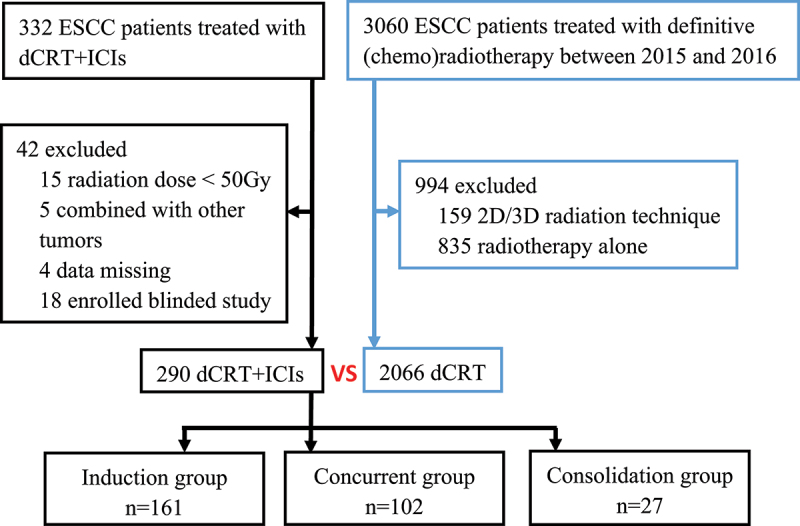
Flow chart depicting patient selection and grouping.

For comparative analysis of the effectiveness of adding ICIs to dCRT, we screened a historical control group from an observational study involving 14 medical centers that investigated the survival outcomes of patients with inoperable ESCC treated with dCRT in China between 2015 and 2016.^6^ Following the flow chart depicted in Figure 1, among a total of 3,060 candidates, 994 were excluded for the following reasons: 159 due to the use of the 2D/3D radiation technique and 835 because they had not received chemotherapy. As a result, a total of 2,066 patients were pooled to comprise the historical control group.

ESCC = Esophageal Squamous Cell Carcinoma, dCRT = definitive chemoradiotherapy, ICIs = Immune check point inhibitors.

This study was approved by the Ethics Committee of the National Cancer Center/Cancer Hospital, Chinese Academy of Medical Sciences, and was conducted in accordance with the 2013 version of the Declaration of Helsinki. Informed consent was waived considering the retrospective nature of the study. The trial is registered with ClinicalTrials.gov (Trial no. NCT04821778).

### Treatment

The peri-radiation period refers to the time frame starting from the initiation of immunotherapy and chemotherapy to six months before radiotherapy to 3 months after radiotherapy. The induction group was defined as a cohort that has completed at least one cycle of immunotherapy before the initiation of radiation. The concurrent group refers to patients who started immunotherapy during the radiotherapy period, while the consolidation group refers to patients who started immunotherapy after radiation.

A taxane-based regimen combined with a platinum-based doublet regimen was the major chemotherapy strategy. The patients received intensity-modulated radiation therapy, volumetric-modulated arc therapy, or helical tomotherapy, all patients underwent conventional fractionated radiotherapy with single-fraction doses ranging from 1.8 to 2.14 Gy. The total radiotherapy duration spanned 35–60 days. Treatment-related adverse events (TRAEs) were graded according to the Common Terminology Criteria for Adverse Events, version 5.0 (US National Institutes of Health, Bethesda, Maryland).

### Statistical analyses

OS was defined as the time interval between the start of initial chemoimmunotherapy or radiotherapy and death of any cause. Data for patients who were alive or lost to follow-up were censored for OS at the time they were last known to be alive. Progression-free survival (PFS) was defined as the time interval between the start of initial therapy and the date of locoregional progression, distant metastases, or death of any cause, whichever occurred first. Data for patients with progression-free or follow-up failure were censored for PFS at the time they were last known without disease progression. Propensity scores were estimated with multinomial logistic regression. The investigated covariates included tumor location, tumor length, T stage, N stage and ICI cycle. The missing covariate values were estimated with multiple imputation. Stabilized inverse probability of treatment weighting (sIPTW) was applied to the estimated propensity scores. To assess balance, standardized mean difference (SMD) in covariate values was compared across treatment groups in an sIPTW sample. An SMD less than 0.1 has been suggested as a cutoff for adequate balance, so this criterion was applied to our sIPTW-adjusted data, too.^22^ Kaplan-Meier curves were used to estimate survival distribution, and duration of follow-up was estimated using the reverse Kaplan-Meier method. An unstratified log-rank test was used to assess inter-group differences in OS and PFS. The Cox proportional hazards model with Efron’s method of tie handling was used to calculate hazard ratios (HRs) and the corresponding 95% confidence intervals (CIs). All statistical analyses were conducted using the R software, version 4.3.1 (R Foundation) and the SPSS software, version 24.0 (IBM Institute Inc). All *p* values were two-tailed, and p < .05 was considered to be statistically significant.

## Results

### Baseline characteristics

Between April 2018 and April 2022, a total of 332 patients were screened at five centers in China, among whom 42 were excluded. The remaining 290 patients were included in the analysis (Figure 1). Among them, 236 (81.4%) patients were male, with a median age of 65 years (range, 40–85 years), and 151 (52.1%) and 108 (37.3%) patients had stage III and IV disease, respectively.

The median radiation dose was 50.4 Gy (range, 50–69.96 Gy), and the median number of immunotherapy cycles was 5 (range, 1–29). The induction group, concurrent group, and consolidation group comprised 161 patients (55.5%), 102 patients (35.2%), and 27 patients (9.3%), respectively. In the induction group, the median number of immune cycles before radiotherapy was 3 (range, 2–8), with a median of 56 days from the start of immunotherapy to the start of radiotherapy (range, 24–180 days). In the consolidation group, the median number of immune cycles was 6 (range, 1–23 days), and the median time from the start of immunotherapy to the end of radiotherapy was 44 days (range, 3–92).

The median number of chemotherapy cycles was 5 (range, 1–19). Pre-radiation chemotherapy was administered in 186 patients (64.1%). During and after radiation, 240 patients (82.8%) and 63 patients (21.7%) received concurrent and consolidation chemotherapy, respectively (Table 1).

**Table 1.** Characteristics of patients (*N* = 290).

Characteristic	N (%)
Median age (range), year	65 (40–85)
Sex	
Male	236 (81.4)
Smoking history	
No	158 (54.5)
Yes	132 (45.5)
Drinking history	
No	162 (55.9)
Yes	128 (44.1)
ECOG score	
0–1	253 (87.2)
2	37 (12.8)
Median BMI (IQR, range)	22.9 (20.8–25.1)
Tumor location	
Upper	128 (44.1)
Middle	119 (41.0)
Lower	43 (14.9)
Median tumor length (IQR, range), cm	5.0 (4.0–7.0)
Clinical T stage	
T1–2	117 (40.3)
T3–4	173 (59.7)
Clinical N stage	
N0	17 (5.9)
N1	89 (30.7)
N2	126 (43.4)
N3	58 (20.0)
Clinical M stage	
M0	250 (86.2)
M1	40 (13.8)
Clinical TNM stage	
I-II	31 (10.6)
III	151 (52.1)
IV	108 (37.3)
ICIs cycle	
1–2	85 (29.3)
3–4	56 (19.3)
5–6	31 (10.7)
≥7	118 (40.7)
Timing of initiation of ICIs	
Before radiation	161 (55.5)
Concurrent radiation	102 (35.2)
After radiation	27 (9.3)
Radiation dosage	
50 Gy	35 (12.1)
>50 Gy	255 (87.9)
Timing of chemotherapy usage	
Before radiation	186 (64.1)
Concurrent radiation	240 (82.8)
After radiation	63 (21.7)

Abbreviation: *IQR* = interquartile range, *BMI* = body mass index, *ECOG* = Eastern Cooperative Oncology Group, *ICIs =* immune checkpoint inhibitors.

### Survival outcomes

Until April 2024, the median follow-up time was 35.7 months (95% CI, 34.5–36.9). The 1-year and 2-year OS rates were 86.7% (95% CI, 82.9–90.8) and 66.9% (95% CI, 61.6–72.6) (Supplementary figure S1A), respectively. The 1-year and 2-year PFS rates were 66.7% (95% CI, 61.5–72.4) and 47.3% (95% CI, 41.9–53.5), respectively (Supplementary figure S1B). The median OS was not reached, and the median PFS was 22.4 months (95% CI, 18.4–26.8).

**Figure 2.**
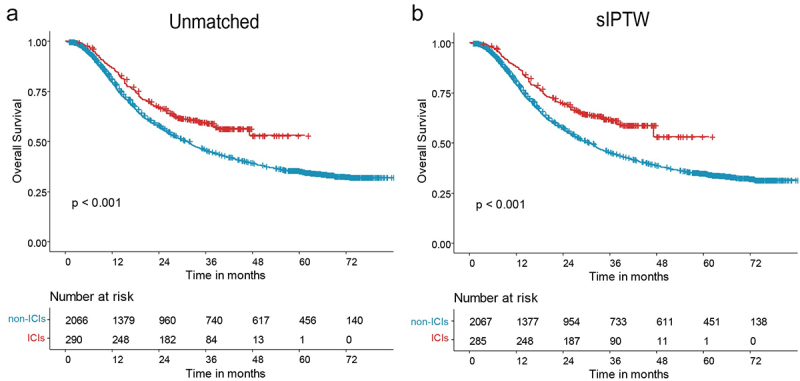
Overall survival data are illustrated in the definitive chemoradiotherapy combined with immune checkpoint inhibitor population compared before (a) and after sIPTW (b).

As compared with the historical control group, the dCRT+ICI group was associated with significantly longer OS (HR = 0.67; 95% CI, 0.56–0.82; *p* < .001; Figure 2a). sIPTW was used to balance age, sex, and clinical TNM stage characteristics between the two groups. The data before and after adjustment with sIPTW are shown in Supplementary table S2 (Supplementary figure S2). The sIPTW-adjusted data indicated that the dCRT+ICI group had significantly better OS than the historical control group (HR = 0.62; 95% CI, 0.50–0.75; *p* < .001) (Figure 2b).

sIPTW=stabilized inverse probability of treatment weighting.

### Univariate and multivariate cox regression analyses of OS and PFS

The results of univariate analyses showed that tumor length ≥5 cm, T3–4, N2–3, and number of ICI cycles ≤ 4 were significantly associated with shorter OS. Further, history of alcohol consumption, T3–4, N2–3, and number of ICI cycles ≤ 4 were significantly associated with poorer PFS (Supplementary table S3). Multivariate analyses confirmed that N2–3 and number of ICI cycles ≤ 4 were independent adverse factors for OS and PFS.

### Effect of immunotherapy intervention time on survival outcomes

**Figure 3.**
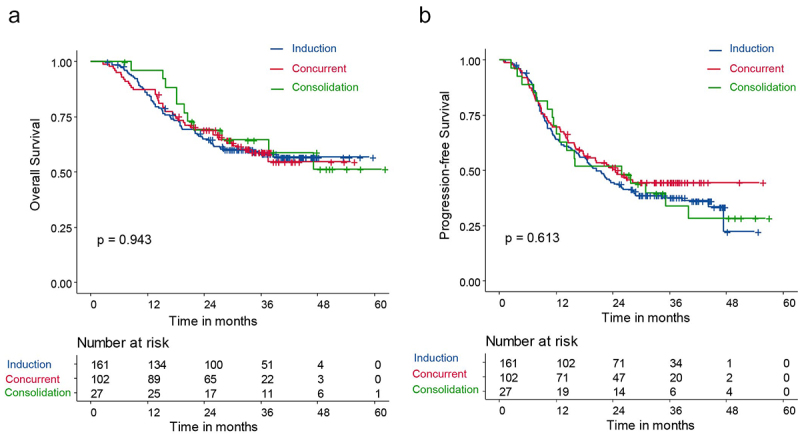
Overall survival and progression-free survival in the induction, concurrent, and consolidation groups (a) overall survival and (b) progression-free survival of patients with inoperable esophageal squamous cell carcinoma after immunotherapy combined with definitive chemoradiotherapy according to the timing of immunotherapy initiation.

According to the first application of immunotherapy before, during, and after radiation, 290 patients were divided into the induction group (161 patients), the concurrent group (102 patients), and the consolidation group (27 patients). The clinical characteristics are shown in Supplementary table S4. The 1- and 2-year OS and PFS did not differ significantly between the three groups. The 1- and 2-year OS were 84.8% (95% CI, 79.4–90.6) and 65.0% (95% CI, 57.9–72.9), respectively, in the induction group; 87.3% (95% CI, 81.0–94.0) and 69.3% (95% CI, 60.9–78.9), respectively, in the concurrent group; 96.2% (95% CI, 89.0–100.0) and 69.0% (95% CI, 53.3–89.4), respectively, in the consolidation group (*p* = .943; Figure 3a). The 1-year and 2-year PFS were 64.2% (95% CI, 57.1–72.1) and 44.7% (95% CI, 37.6–53.1), respectively, in the induction group; 69.6% (95% CI, 61.2–79.1) and 50.4% (95% CI, 41.5–61.2), respectively, in the concurrent group; and 70.4% (95% CI, 55.1–89.9) and 51.9% (95% CI, 36.1–74.6), respectively, in the consolidation group (*p* = .613; Figure 3b). The median OS was not reached in all three groups, and median PFS was 20.5 months (95% CI, 16.9–26.1), 24.6 months (95% CI, 16.8–NR), and 25.8 months (95% CI, 12.8–NR) in the induction, concurrent, and consolidation groups respectively.

sIPTW was applied to balance the clinical characteristics of the induction group, concurrent group, and consolidation group. The baseline characteristics of patients before and after sIPTW adjustment are shown in Supplementary tables S5–7. The sIPTW-adjusted data showed that the PFS of both the induction group and the concurrent group was significantly longer than that of the consolidation group (induction vs. consolidation: HR = 0.441, *p* = .023; concurrent vs. consolidation: HR = 0.429, *p* = .019; Supplementary figures S3–5). However, no difference was observed between the induction and concurrent groups.

### Treatment-related adverse events

A total of 119 (119/290, 41.0%) patients had recorded TRAEs, including 33.5% (54/161) patients in the induction group, 55.9% (57/102) patients in the concurrent group, and 29.6% (8/27) patients in the consolidation group (Table 2). The TRAEs with an incidence rate > 20% were leukopenia (81.5%), esophagitis (74.8%), neutropenia (71.4%), pruritus (44.5%), rash (43.7%), and hypothyroidism (21.0%). Grade 3–4 TRAEs, which occurred in > 10% of the whole cohort, included leukopenia (17.6%), esophagitis (12.6%), and neutropenia (10.9%). The most common grade 3–4 TRAEs in the induction group were leukopenia (25.9%) and neutropenia (20.4%). In the concurrent group, the most common grade 3–4 TRAEs were esophagitis (22.8%) and leukopenia (12.3%), while in the consolidation group, pruritus (12.5%) and hypothyroidism (12.5%) were the most prevalent grade 3–4 TRAEs. The incidence rate of pneumonia, which is considered a grade 3–4 TRAE was 3.7%, 8.8%, and 0% in the induction, concurrent, and consolidation groups, respectively. Among the adverse events observed, hypothyroidism, hyperthyroidism, hyperglycemia, rash, and elevated amylase levels were considered immune-related adverse events. There were no treatment-related deaths.

**Table 2.** Treatment-related adverse events.

Treatment-related adverse events	Total *n* = 119 (%)	Induction group *n* = 54 (%)	Concurrent group *n* = 57 (%)	Consolidation group *n* = 8 (%)
		Grade 3–4		
Leukopenia	97 (81.5)	14 (25.9)	7 (12.3)	0 (0.0)
Esophagitis	89 (74.8)	2 (3.7)	13 (22.8)	0 (0.0)
Neutropenia	85 (71.4)	11 (20.4)	2 (3.5)	0 (0.0)
Pruritus	53 (44.5)	1 (1.9)	3 (5.3)	1 (12.5)
Rash*	52 (43.7)	2 (3.7)	0 (0.0)	0 (0.0)
Hypothyroidism*	25 (21.0)	0 (0.0)	3 (5.3)	1 (12.5)
Hyperthyroidism*	20 (16.8)	1 (1.9)	2 (3.5)	0 (0.0)
Pneumonitis	18 (15.1)	2 (3.7)	5 (8.8)	0 (0.0)
Hepatitis	5 (4.2)	0 (0.0)	0 (0.0)	0 (0.0)
Fever	15 (12.6)	1 (1.9)	1 (1.8)	0 (0.0)
Elevation in amylase*	13 (10.9)	0 (0.0)	0 (0.0)	0 (0.0)
Anorexia	10 (8.4)	0 (0.0)	0 (0.0)	0 (0.0)
Colitis	8 (6.7)	0 (0.0)	0 (0.0)	0 (0.0)
Elevation in lipase*	8 (6.7)	0 (0.0)	0 (0.0)	0 (0.0)
Arthralgia	7 (5.9)	0 (0.0)	0 (0.0)	0 (0.0)
Myalgia	7 (5.9)	0 (0.0)	0 (0.0)	0 (0.0)
Hepatitis	5 (4.2)	0 (0.0)	0 (0.0)	0 (0.0)
Fatigue	2 (1.7)	0 (0.0)	0 (0.0)	0 (0.0)
Adrenal insufficiency*	2 (1.7)	0 (0.0)	0 (0.0)	0 (0.0)

* Considered immune-related adverse events.

### Patterns of recurrence

At the time of analysis, the site of recurrence was known in 134 patients (46.2%), including 81 patients (27.9%) who had locoregional progression, 53 patients (18.3%) who had distant metastasis, and 12 patients (4.1%) who had both. Distant metastases were most frequent in the lungs (25 cases, 8.6%) and liver (13 cases, 4.5%) (Table 3).

**Table 3.** Pattern of first recurrence sites.

First site of recurrence	Patients with recurrent disease, *n* = 290, n (%)
Locoregional	93 (27.9)
Distant	53 (18.3)
Lung	25 (8.6)
Liver	13 (4.5)
Bone	7 (2.4)
Other sites	11 (3.8)
Both	12 (4.1)

## Discussion

To our knowledge, this study is the first multicenter real-world study on the efficacy of dCRT+ICIs for inoperable ESCC. Our findings reveal that the addition of ICIs to dCRT, as compared to treatment with dCRT alone (based on data from a historical control group), improves OS while offering a favorable safety profile for patients with inoperable ESCC. With regard to the timing of immunotherapy, after sIPTW adjustment, the PFS in the induction and concurrent groups was found to be superior to that in the consolidation group. Thus, it appears that adding immunotherapy before or during dCRT is more beneficial than applying immunotherapy as consolidation treatment after the dCRT course.

Several other researchers have explored the role of dCRT combined with immunotherapy in inoperable esophageal cancer. For example, a phase Ib study of concurrent dCRT and sequential camrelizumab in the treatment of LA-ESCC reported that the 2-year OS was of 69.6%.^12^ Further, Park et al. reported that the 2-year OS for patients with locally advanced and postoperative locally recurrent ESCC treated with concurrent and sequential durvalumab and tremelimumab combined with dCRT was 75%, compared to 59.2% in the historical control group treated with only dCRT.^13^ Recently, Ai et al. indicated that induction chemoimmunotherapy followed by concurrent chemoradiotherapy generated a surprisingly promising 2-year OS of 77.2% and a model-based estimation of median OS of 80 months in a prospective phase II study on the treatment of LA-ESCC.^17^ The above findings suggest that the addition of immunotherapy to dCRT can improve survival outcomes. Along the lines of these studies, our study also demonstrated that adding immunotherapy during the peri-dCRT period resulted in a 2-year OS of 66.9%, which is higher than the 2-year OS of 56.5% after dCRT alone in the historical control group. However, a recent prospective phase II study (EC-CRT-001) that included 42 patients did not find any improvement in survival outcome on treatment with a combination of immunotherapy and dCRT. The survival curves showed a 2-year OS rate of approximately 50%, which was lower than that in both the present study and our historical control group.^14^ This may be related to higher proportions of T3–4 (71% vs. 59.7%) and N2–3 (81% vs. 63.4%) cancers in the EC-CRT-001 study. Additionally, the median follow-up time in that study was 14.9 months; thus, the 2-year OS survival rate needs further adjustment with longer follow-up.

Several studies have shown that the locoregional recurrence rate of ESCC after dCRT is 42.9–50.7%, and the distant metastasis rate is 27.5–48.0%.^23–25^ A few dCRT+ICI studies have analyzed recurrence after treatment. For example, Park’s study demonstrated that the dCRT+ICI group exhibited a reduction in both locoregional recurrence (20% vs. 33.3%) and distant metastasis rates (15.0% vs. 45.3%) compared to the group treated with only dCRT.^13^ In the present study, 32.0% of the patients developed locoregional recurrence, and 22.4% developed distant metastasis. Unfortunately, in the present study, we were unable to compare locoregional recurrence because data on disease progression were not available for our historical control group. Moreover, it should be noted that both our study and Park’s study have a median follow-up time of less than 3 years. Therefore, future research should include longer follow-up periods and larger sample sizes to further strengthen our findings and clarify whether immunotherapy can indeed alter the recurrence pattern.

The optimal treatment sequence of immunotherapy combined with radiation remains unclear. The PACIFIC trial (dCRT followed by durvalumab for the treatment of stage III NSCLC) established a basis for consolidative immunotherapy in unresectable NSCLC.^26^ Some research has indicated that concurrent dCRT at different time points may result in variations in peripheral blood tumor-reactive CD8+ T-cells. That is, while the frequency of tumor-reactive CD8+ T-cells increased during dCRT and peaked in the last week of radiotherapy, it decreased at 1 month after dCRT.^27^ Subanalyses of the PACIFIC trial suggest that earlier administration of durvalumab, within 14 days after completion of dCRT, may confer greater survival benefits than later administration.^26^ This indicates the potential benefits of initiating immunotherapy concurrently with radiation or early consolidation.^27^ In the consolidation group of our study, the median time from the last radiotherapy day to the initiation of immunotherapy was 44 days, and this group exhibited poorer PFS than the induction and concurrent immunotherapy groups. This further supports the previous findings and, further, implies that delayed immunotherapy after radiotherapy may result in a reduced synergistic effect. However, the small sample size in our consolidation group may limit the persuasive power of the findings.

The definitive radiotherapy target of esophageal cancer typically includes a long length of primary tumor and multiple lymph node metastatic areas such as the supraclavicular, mediastinal, and abdominal regions. Extended exposure to moderate doses over a larger irradiation field could have detrimental effects on the immune system, as it may result in the clearance of effector cell types crucial for potent antitumor activity.^28^ Additionally, concurrent dCRT+ICI may lead to increased toxicity. This may explain why concurrent and consolidative durvalumab with dCRT did not result in better survival than dCRT alone in the PACIFIC-2 study on the treatment of stage III NSCLC.^15^ With regard to esophageal cancer, randomized phase III studies that confirm the sequence of immunotherapy combined with radiotherapy are still lacking. Based on our findings, induction chemoimmunotherapy may be the optimal strategy because tumor regression following induction therapy may facilitate smaller radiation target volumes, provide improved protection of critical organs, and minimize lymphocyte damage. Additionally, Ai et al. found that induction chemoimmunotherapy can promote vascular normalization and alleviate hypoxia in esophageal cancer, thus increasing radiotherapy sensitivity and improving patient outcomes.^17^ These findings suggest that early administration of immunotherapy before radiotherapy may provide greater survival benefits for inoperable LA-ESCC. In our study, the improvement in PFS was greater in the induction group than in the consolidation group even after sIPTW, further supporting the benefits of induction immunotherapy in this group of patients. While the current findings point to induction chemotherapy being more beneficial than other sequences, in the future, prospective clinical trials are required to validate the optimal sequence of immunotherapy combined with radiotherapy in esophageal cancer.

In this study, the incidence of the TRAEs esophagitis and pneumonitis of any grade in the overall population was 74.8% and 15.1%, respectively, and the rates of grade 3 and 4 events was 12.6% and 5.9%, respectively. In Zhang’s study, the incidence of ≥grade 3 esophagitis and pneumonitis was 20% and 4%, respectively, and in the EC-CRT-001 study, the incidence was 10% and 6.1%, respectively.^12,14^ Thus, their findings are similar to those of our study. Our results also showed that the proportion of grade 3–4 leukopenia and neutropenia cases in the induction group (25.9% and 20.4%, respectively) was significantly higher than that in the concurrent group (12.3% and 3.5%, respectively) and the consolidation group (0% and 0%, respectively). Thus, these outcomes may be related to severe myelosuppression and impaired hematopoietic reserve following induction therapy. Additionally, this study observed that the incidence of esophagitis and pneumonitis was significantly higher in the concurrent group, at 22.8% and 8.8%, respectively, compared to the induction group (3.7% and 3.7%, respectively) and the consolidation group (0% and 0%, respectively). This is probably attributable to the cumulative toxicity associated with synchronous treatment modalities.

This study has several limitations, Firstly, it is a retrospective analysis in which the data collection method may have introduced an inherent population bias. Moreover, the findings are also limited by missing data. Some other limitations are a lack of clear delineation of the target volume and differences in chemotherapy regimens and PD-1 drugs (as they were from different manufacturers). Further, the sample size in the consolidation immunotherapy group was smaller than that of the other two groups. Finally, PD-L1 expression data were missing, and this precluded analysis of the relationship between PD-L1 expression and survival outcomes. However, several large randomized controlled trials on advanced ESCC have shown similar efficacy benefits from immune combination therapy across different PD-L1 expression subgroups.^11,29,30^ PD-L1 testing is currently not routine in China for patients with ESCC before the application of immunotherapy.

In summary, the findings of the current study suggest that combining immunotherapy with dCRT has favorable efficacy and safety in patients with inoperable ESCC. With regard to the treatment sequence, early administration of immunotherapy before or concurrent with radiotherapy showed improved PFS benefits.

## Supplementary Material

Supplementary_figure_1.jpg

Supplementary_figure_4.jpg

Supplementary_figure_3.jpg

Supplementary_figure_5.jpg

Supplementary Tables.docx

Supplementary_figure_2.jpg

## Data Availability

The data that support the findings of this study are available from the corresponding author, Xin Wang, upon reasonable request.
