# Supplementary figures and images for: Definitive chemoradiotherapy combined with anti-PD-1 immunotherapy for inoperable esophageal squamous cell carcinoma: a multicenter real-world study
Source: Cancer Biol Ther. 2025 May 14;26(1):2504726. doi: 10.1080/15384047.2025.2504726 (PMC12080274; doi:10.1080/15384047.2025.2504726)

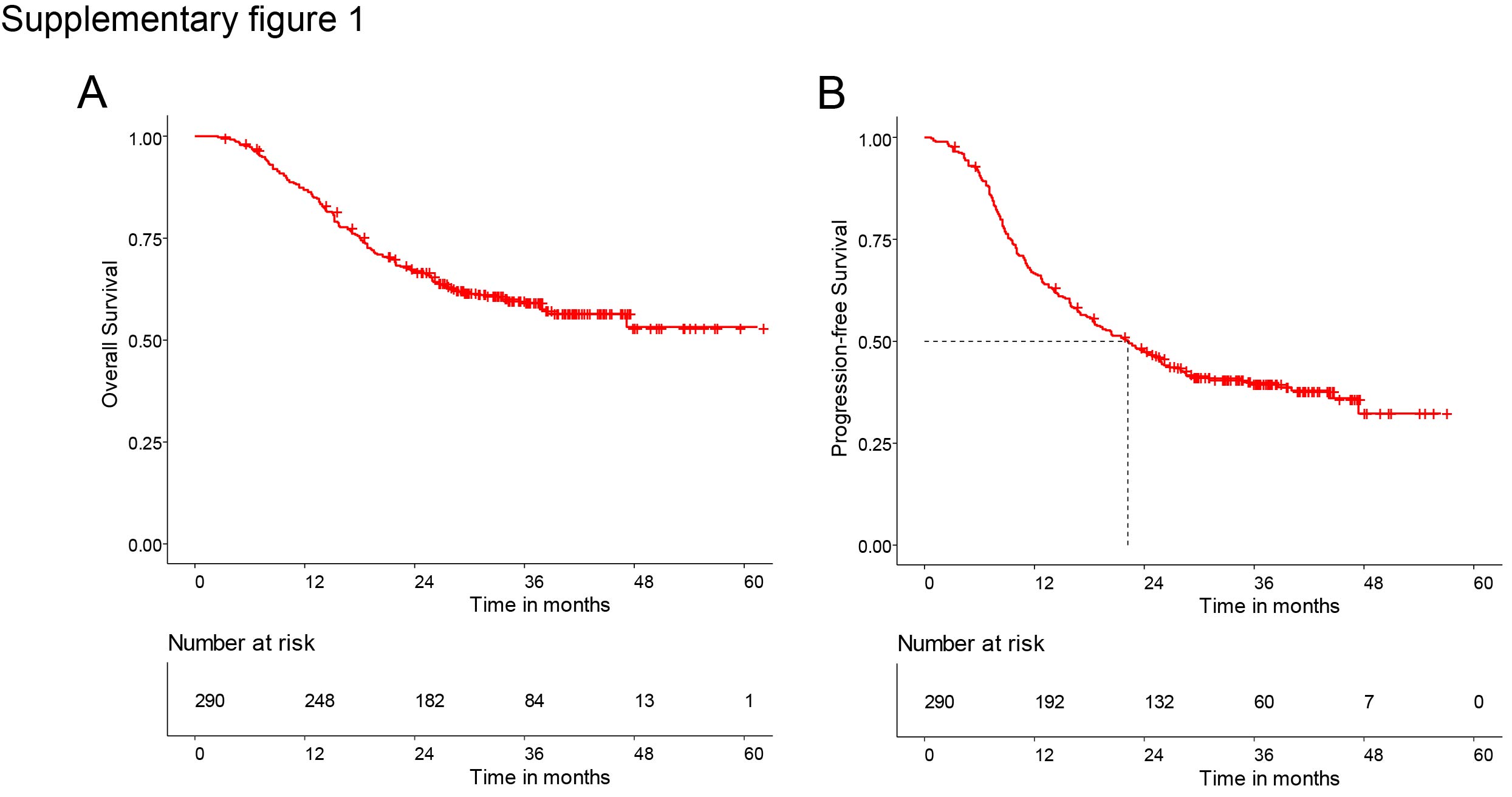

Supplement: Supplementary_figure_1.jpg [file KCBT_A_2504726_SM7574.jpg]

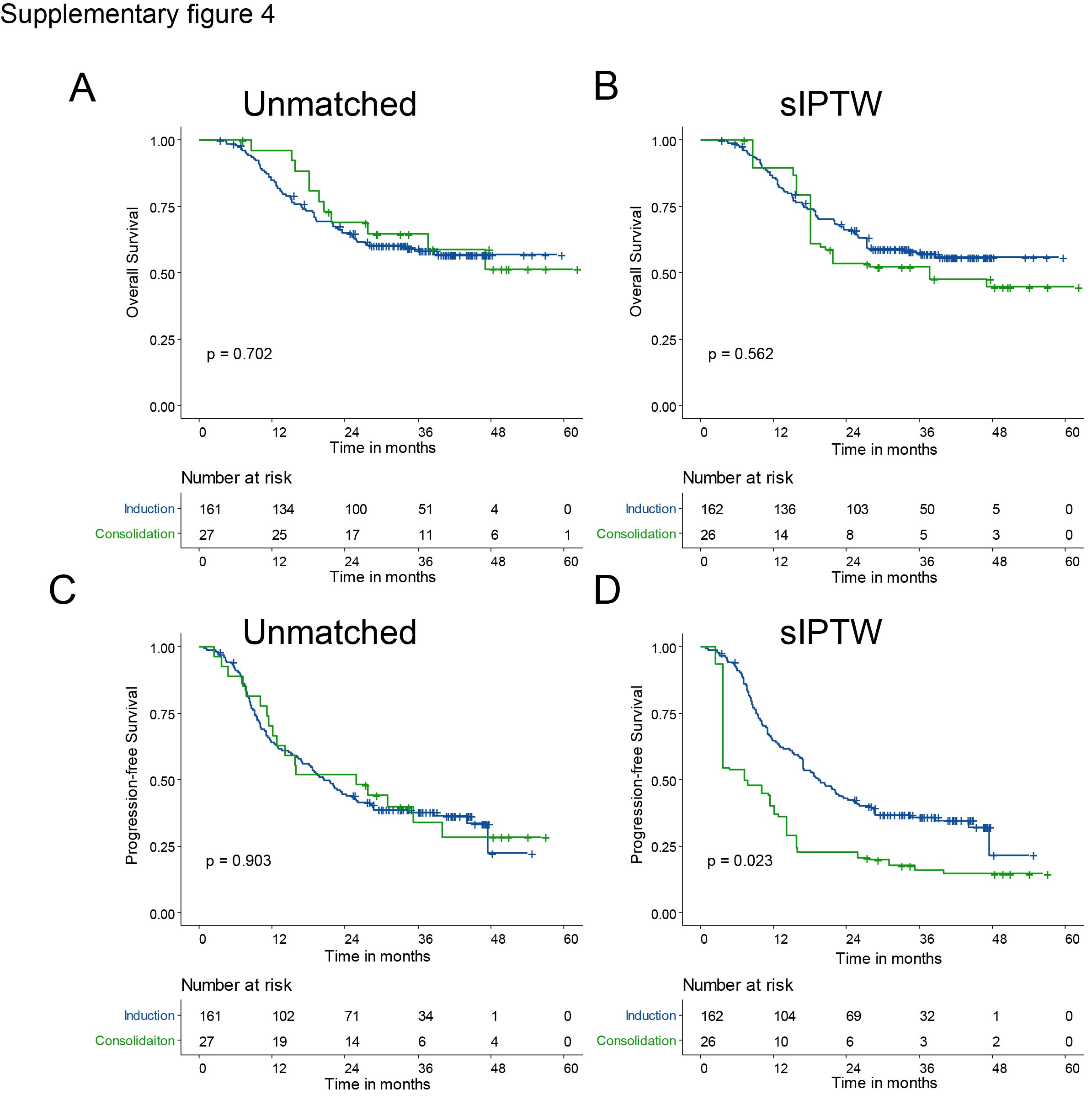

Supplement: Supplementary_figure_4.jpg [file KCBT_A_2504726_SM7573.jpg]

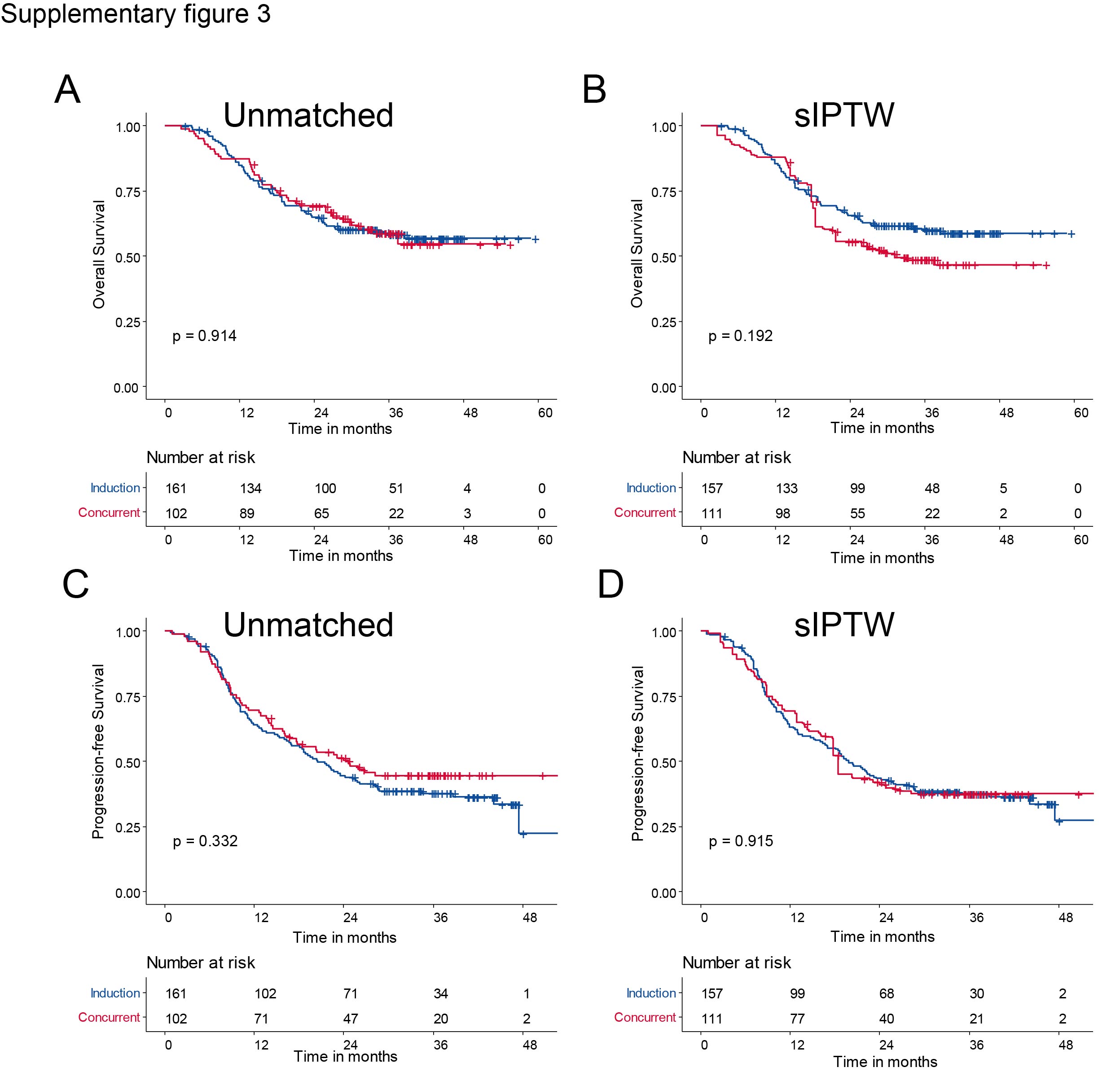

Supplement: Supplementary_figure_3.jpg [file KCBT_A_2504726_SM7572.jpg]

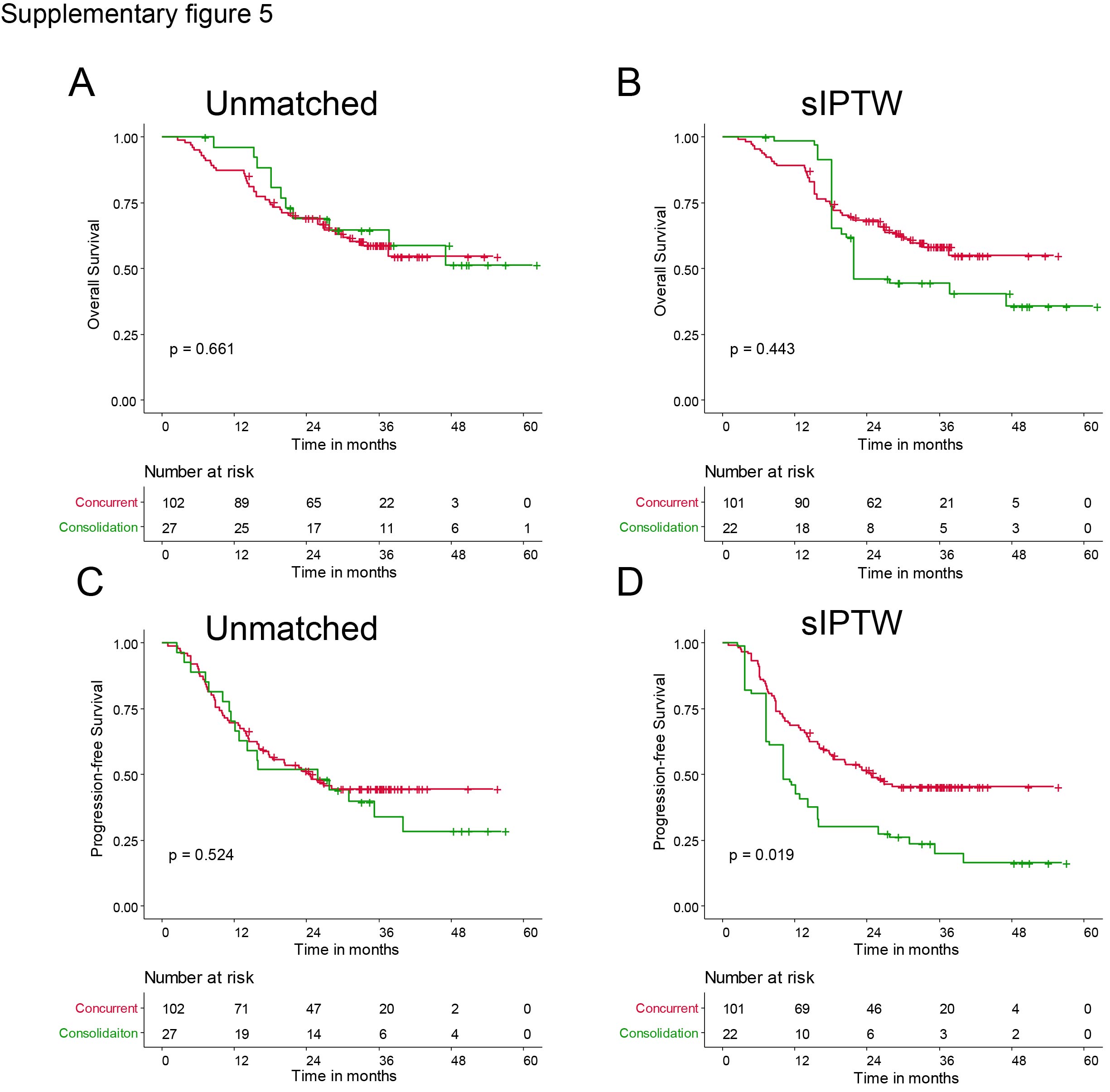

Supplement: Supplementary_figure_5.jpg [file KCBT_A_2504726_SM7571.jpg]

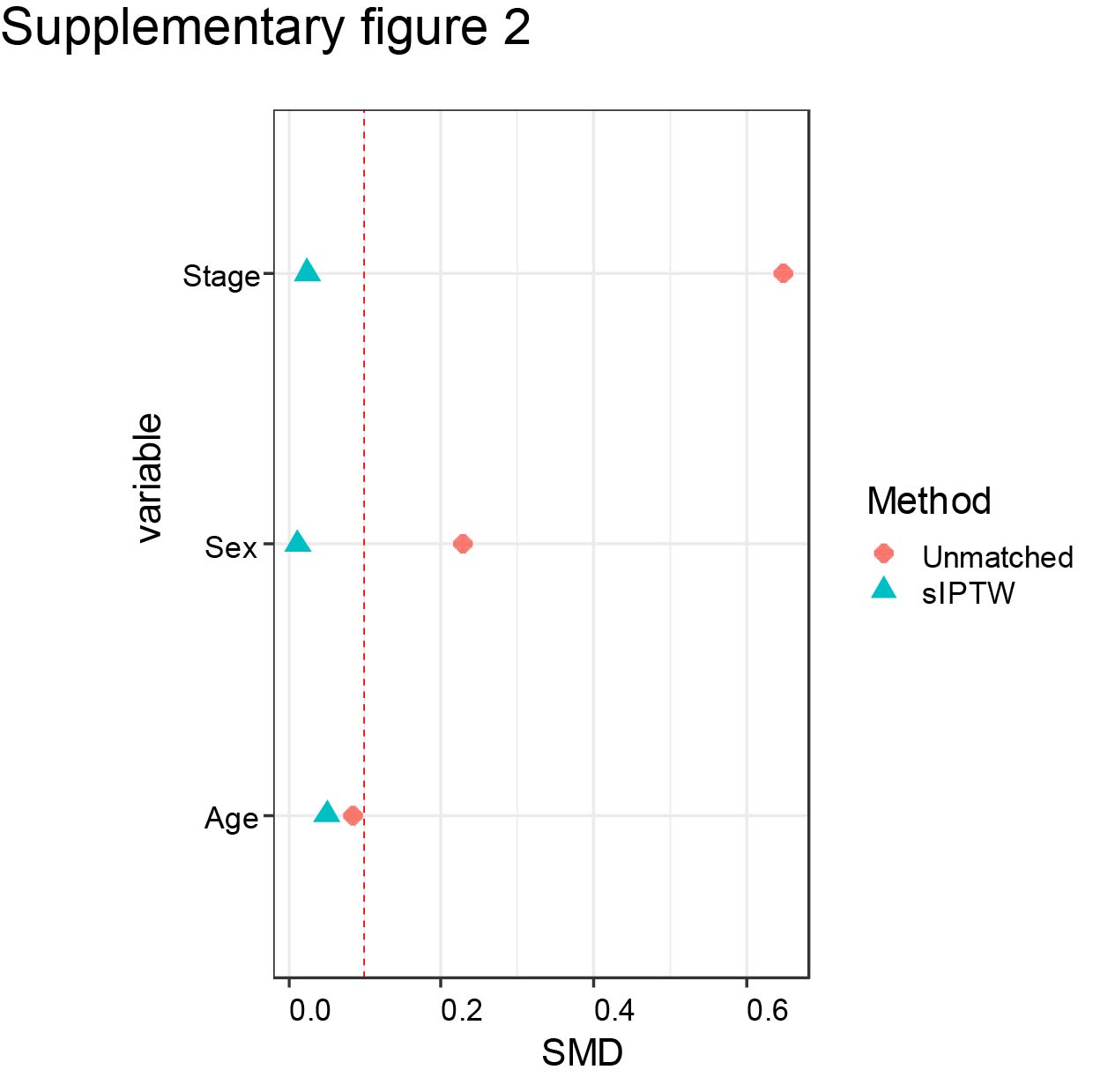

Supplement: Supplementary_figure_2.jpg [file KCBT_A_2504726_SM7569.jpg]
