# Supplementary material for: Definitive chemoradiotherapy combined with anti-PD-1 immunotherapy for inoperable esophageal squamous cell carcinoma: a multicenter real-world study
Source: Cancer Biol Ther. 2025 May 14;26(1):2504726. doi: 10.1080/15384047.2025.2504726 (PMC12080274; doi:10.1080/15384047.2025.2504726)
Supplement: Supplementary Tables.docx [file KCBT_A_2504726_SM7570.docx]

Supplementary Table1: Name of participating medical center and number of enrolled cases.

| Medical center | Cases |
| --- | --- |
| National Cancer Center/National Clinical Research Center for Cancer/Cancer Hospital, Chinese Academy of Medical Sciences and Peking Union Medical College | 74 |
| The Affiliated Cancer Hospital of Nanjing Medical University, Jiangsu Cancer Hospital, Jiangsu Institute of Cancer Research | 128 |
| Anyang Cancer Hospital and The Affiliated Anyang Cancer Hospital of Henan University of Science and Technology | 42 |
| Hunan Cancer Hospital and The Affiliated Cancer Hospital of Xiangya School of Medicine | 34 |
| Jiangsu Province Hospital and Nanjing Medical University First Affiliated Hospital | 12 |

Supplementary Table 2: Patient characteristics (Unmatched and sIPTW-adjusted) in historical control group and definitive chemoradiotherapy combined with immune checkpoint inhibitors groups.

| Group | Unmatched | | | sIPTW | | |
| --- | --- | --- | --- | --- | --- | --- |
|  | Historical control group | dCRT+ICIs | P | historical control group | dCRT+ICIs | P |
| N | 2066 | 290 |  | 2067 | 285 |  |
| Age (mean, SD) | 64.6 (8.5) | 63.9 (7.9) | 0.192 | 64.5 (8.6) | 64.1 (7.8) | 0.481 |
| Sex (%) |  |  | 0.001 |  |  | 0.887 |
| Male | 1483 (71.8) | 236 (81.4) |  | 1507(72.9) | 210 (73.7) |  |
| Female | 583 (28.2) | 54 (18.6) |  | 560 (27.1) | 75 (26.3) |  |
| TNM stage (%) |  |  | <0.001 |  |  | 0.994 |
| I | 56 ( 2.7) | 1 ( 0.3) |  | 50 ( 2.4) | 6 ( 2.1) |  |
| II | 615 (29.8) | 30 ( 10.3) |  | 563 (27.2) | 81 (28.4) |  |
| III | 848 (41.0) | 151 (52.1) |  | 878 (42.5) | 123 (43.2) |  |
| IVa | 268 (13.0) | 76(26.3) |  | 302 (14.6) | 43 (15.1) |  |
| IVb | 279 (13.5) | 32 (11.0) |  | 274 (13.3) | 32 (11.2) |  |

Abbreviation: s*IPTW* = stabilized inverse probability of treatment weighting, *dCRT* = definitive chemoradiotherapy, *ICIs* = immune checkpoint inhibitors, *SD* = standard deviation.

Supplementary Table 3: Univariate and stepwise multivariate Cox hazard analysis of risk factors for overall survival and progression-free survival.

| Characteristics | OS | | | | PFS | | | |
| --- | --- | --- | --- | --- | --- | --- | --- | --- |
|  | Univariate analysis | | Multivariate analysis | | Univariate analysis | | Multivariate analysis | |
|  | HR (95% CI) | p-Value | HR (95% CI) | p-Value | HR (95% CI) | p-Value | HR (95% CI) | p-Value |
| Age |  |  |  |  |  |  |  |  |
| < 65 | Ref |  |  |  | Ref |  |  |  |
| ≥ 65 | 1.269  (0.879-1.832) | 0.203 |  |  | 0.951  (0.706-1.280) | 0.739 |  |  |
| Sex |  |  |  |  |  |  |  |  |
| Female | Ref |  |  |  | Ref |  |  |  |
| Male | 1.685  (0.994-2.857) | 0.053 |  |  | 1.495  (0.992-2.255) | 0.055 |  |  |
| Smoking history |  |  |  |  |  |  |  |  |
| No | Ref |  |  |  | Ref |  |  |  |
| Yes | 1.363  (0.945-1.966) | 0.097 |  |  | 1.292  (0.958-1.741) | 0.093 |  |  |
| Drinking history |  |  |  |  |  |  |  |  |
| No | Ref |  |  |  | Ref |  | Ref |  |
| Yes | 1.340  (0.929-1.933) | 0.117 |  |  | 1.431  (1.062-1.930) | **0.019** | 1.417  (0.896-2.241) | 0.136 |
| ECOG score |  |  |  |  |  |  |  |  |
| 0-1 | Ref |  |  |  | Ref |  |  |  |
| 2 | 1.248  (0.847-1.839) | 0.262 |  |  | 1.245  (0.909-1.707) | 0.173 |  |  |
| BMI |  |  |  |  |  |  |  |  |
| < 23.0 | Ref |  |  |  | Ref |  |  |  |
| ≥ 23.0 | 1.098  (0.760-1.586) | 0.620 |  |  | 0.811  (0.600-1.097) | 0.175 |  |  |
| Tumor location |  |  |  |  |  |  |  |  |
| Upper | Ref |  |  |  | Ref |  |  |  |
| Middle | 1.235  (0.837-1.821) | 0.287 |  |  | 1.019  (0.743-1.399) | 0.906 |  |  |
| Lower | 0.828  (0.457-1.500) | 0.534 |  |  | 0.646  (0.396-1.054) | 0.080 |  |  |
| Tumor length (cm) |  |  |  |  |  |  |  |  |
| < 5 | Ref |  | Ref |  | Ref |  |  |  |
| ≥ 5 | 1.521  (1.010-2.289) | **0.045** | 1.513  (0.990-2.313) | 0.056 | 1.356  (0.978-1.881) | 0.068 |  |  |
| Clinical T stage |  |  |  |  |  |  |  |  |
| T1-2 | Ref |  | Ref |  | Ref |  | Ref |  |
| T3-4 | 1.793  (1.204-2.670) | **0.004** | 1.388  (0.903-2.134) | 0.135 | 1.626  (1.184-2.232) | **0.003** | 1.337  (0.944-1.894) | 0.101 |
| Clinical N stage |  |  |  |  |  |  |  |  |
| N0-1 | Ref |  | Ref |  | Ref |  | Ref |  |
| N2-3 | 1.920  (1.271-2.900) | **0.002** | 1.812  (1.138-2.883) | **0.012** | 1.702  (1.228-2.360) | **0.001** | 1.703  (1.171-2.477) | **0.005** |
| Clinical M stage |  |  |  |  |  |  |  |  |
| M0 | Ref |  |  |  | Ref |  |  |  |
| M1 | 0.724  (0.389-1.348) | 0.309 |  |  | 0.781  (0.485-1.258) | 0.310 |  |  |
| ICIs cycle |  |  |  |  |  |  |  |  |
| ≤ 4 | Ref |  | Ref |  | Ref |  | Ref |  |
| > 4 | 0.610  (0.422-0.881) | **0.008** | 0.589  (0.401-0.865) | **0.007** | 0.677  (0.502-0.913) | **0.011** | 0.637  (0.464-0.876) | **0.006** |
| Radiation dosage |  |  |  |  |  |  |  |  |
| 50Gy | Ref |  |  |  | Ref |  |  |  |
| ≥ 50Gy | 0.897  (0.521-1.544) | 0.694 |  |  | 0.996  (0.636-1.558) | 0.985 |  |  |

Abbreviation: *BMI* = body mass index, *ECOG* = Eastern Cooerpative Oncology Group, *ICIs =* immune checkpoint inhibitors, *HR* = hazard analysis, *CI*=confidence interval.

Supplementary Table 4:Baseline characteristics of patients among the three groups.

| Characteristic | Induction group  (N=161) | Concurrent group  (N=102) | Consolidation group  (N=27) |
| --- | --- | --- | --- |
| Median age (range), years | 64 (40-84) | 66 (46-82) | 61 (47-85) |
| Sex (%) |  |  |  |
| Male | 133 (82.6） | 81 (79.4） | 22 (81.5） |
| Female | 28 (17.4) | 21 (20.6) | 5 (18.5) |
| Smoking history (%) |  |  |  |
| No | 92 (57.1) | 45 (44.1) | 21 (77.8) |
| Yes | 69 (42.9) | 57 (55.9) | 6 (22.2) |
| Drinking history (%) |  |  |  |
| No | 94 (58.4) | 50 (49.0) | 18 (66.7) |
| Yes | 67 (41.6) | 52 (51.0) | 9 (33.3) |
| ECOG score (%) |  |  |  |
| 0-1 | 144 (89.4) | 90 (88.2) | 19 (70.4) |
| 2 | 17 (10.6) | 12 (11.8) | 8 (29.6) |
| Median BMI (IQR) | 22.9 (20.8-25.6) | 22.8 (20.7-24.8) | 23.3 (21.3-25.9) |
| Tumor location (%) |  |  |  |
| Upper | 73 (45.3) | 41 (40.2) | 14 (51.9) |
| Middle | 59 (36.6) | 52 (51.0) | 8 (29.6) |
| Lower | 29 (18.1) | 9 (8.8) | 5 (18.5) |
| Median tumor length (IQR), cm | 5.0 (4.0-7.0) | 5.4 (4.6-7.0) | 6.0 (4.0-9.0) |
| Clinical T stage (%) |  |  |  |
| T1-2 | 55 (34.2) | 54 (52.9) | 8 (29.6) |
| T3-4 | 106 (65.8) | 48 (47.1) | 19 (70.4) |
| Clinical N stage (%) |  |  |  |
| N0 | 5 (3.1) | 11 (10.8) | 1 (3.7) |
| N1 | 44 (27.3) | 38 (37.3) | 7 (25.9) |
| N2 | 75 (46.6) | 39 (38.2) | 12 (44.4) |
| N3 | 37 (23.0) | 14 (13.7) | 7 (26.0) |
| Clinical M stage (%) |  |  |  |
| M0 | 139 (86.3) | 90 (88.2) | 21 (77.8) |
| M1 | 22 (13.7) | 12 (11.8) | 6 (22.2) |
| Clinical TNM stage (%) |  |  |  |
| I-II | 12 (7.4) | 16 (15.7) | 3 (11.2) |
| III | 86 (53.4) | 52 (51.0) | 13 (48.1) |
| IV | 63 (39.2) | 34 (33.3) | 11 (40.7) |
| ICIs cycle (%) |  |  |  |
| 1-2 | 69 (42.9) | 11 (10.8) | 5 (18.5) |
| 3-4 | 32 (19.9) | 19 (18.6) | 5 (18.5) |
| 5-6 | 16 (9.9) | 12 (11.8) | 3 (11.1) |
| ≥ 7 | 44 (27.3) | 60 (58.8) | 14 (51.9) |

Abbreviation: *IQR* = interquartile range, *BMI* = body mass index, *ECOG* = Eastern Cooerpative Oncology Group, *ICIs =* immune checkpoint inhibitors.

Supplementary Table 5: Patient characteristics in induction and concurrent groups before and after sIPTW-adjustment.

| Group | Unmatched | | | sIPTW | | |
| --- | --- | --- | --- | --- | --- | --- |
|  | Induction group | Concurrent group | P | Induction group | Concurrent group | P |
| N | 161 | 102 |  | 157 | 111 |  |
| Age (mean (SD)), years | 63.6 (7.4) | 64.2 (7.8) | 0.583 | 63.4 (7.4) | 64.2 (7.8) | 0.553 |
| Sex (%) |  |  | 0.627 |  |  | 0.562 |
| Male | 133 (82.6） | 81 (79.4） |  | 131 (83.6) | 89 (79.9) |  |
| Female | 28 (17.4) | 21 (20.6) |  | 26 (16.4) | 22 (20.1) |  |
| Smoking history (%) |  |  | 0.053 |  |  | 0.790 |
| No | 92 (57.1) | 45 (44.1) |  | 80 (50.8) | 54 (48.5) |  |
| Yes | 69 (42.9) | 57 (55.9) |  | 77 (49.2) | 57 (51.5) |  |
| Drinking history (%) |  |  | 0.174 |  |  | 0.684 |
| No | 94 (58.4) | 50 (49.0) |  | 83 (52.9) | 62 (56.2) |  |
| Yes | 67 (41.6) | 52 (51.0) |  | 74 (47.1) | 49 (43.8) |  |
| ECOG score (%) |  |  | 0.026 |  |  | 0.842 |
| 0-1 | 144 (89.4) | 90 (88.2) |  | 140 (88.9) | 98 (88.3) |  |
| 2 | 17 (10.6) | 12 (11.8) |  | 17 (11.1) | 13 (11.7) |  |
| BMI (mean (SD)) | 23.1 (3.5) | 22.9 (3.2) | 0.786 | 23.0 (3.4) | 23.3 (3.2) | 0.612 |
| Tumor location (%) |  |  | 0.039 |  |  | 0.947 |
| Upper | 73 (45.3) | 41 (40.2) |  | 70 (44.6) | 47 (42.3) |  |
| Middle | 59 (36.6) | 52 (51.0) |  | 65 (41.3) | 45 (40.7) |  |
| Lower | 29 (18.1) | 9 (8.8) |  | 22 (12.9) | 19 (17.0) |  |
| Tumor length (mean (SD)), cm | 5.5 (2.9) | 5.8 (2.2) | 0.344 | 5.6 (3.1) | 5.5 (1.9) | 0.773 |
| Clinical T stage (%) |  |  | 0.009 |  |  | 0.797 |
| T1-2 | 55 (34.2) | 54 (52.9) |  | 58 (36.9) | 36 (32.4) |  |
| T3-4 | 106 (65.8) | 48 (47.1) |  | 99 (63.1) | 75 (67.6) |  |
| Clinical N stage (%) |  |  | 0.037 |  |  | 0.970 |
| N0 | 5 (3.1) | 11 (10.8) |  | 10 ( 6.4) | 6 ( 5.6) |  |
| N+ | 156 (96.9) | 91 (89.2) |  | 147 (93.6) | 105 (94.4) |  |
| Clinical M stage (%) |  |  | 0.821 |  |  | 0.306 |
| M0 | 139 (86.3) | 90 (88.2) |  | 132 (84.2) | 82 (73.8) |  |
| M1 | 22 (13.7) | 12 (11.8) |  | 25 (15.8) | 29 (26.2) |  |
| Clinical TNM stage (%) |  |  | 0.106 |  |  | 0.981 |
| I-II | 12 (7.4) | 16 (15.7) |  | 15 ( 9.4) | 11 (10.3) |  |
| III-IV | 149 (92.6) | 86 (84.3) |  | 142 (90.6) | 100 (89.7) |  |
| ICIs cycle (%) |  |  | <0.001 |  |  | 0.885 |
| 1-2 | 69 (42.9) | 11 (10.8) |  | 51 (32.3) | 43 (39.0) |  |
| 3-4 | 32 (19.9) | 19 (18.6) |  | 31 (19.6) | 20 (17.7) |  |
| 5-6 | 16 (9.9) | 12 (11.8) |  | 17 (10.7) | 11 ( 9.4) |  |
| ≥ 7 | 44 (27.3) | 60 (58.8) |  | 58 (37.4) | 37 (33.9) |  |

Abbreviation: *BMI* = body mass index, *ECOG* = Eastern Cooerpative Oncology Group, *ICIs =* immune checkpoint inhibitors, *SD* = standard deviation, *sIPTW* = stabilized inverse probability of treatment weighting.

Supplementary Table 6: Patient characteristics in induction and consolidation groups before and after sIPTW-adjustment.

| Group | Unmatched | | | sIPTW | | |
| --- | --- | --- | --- | --- | --- | --- |
|  | Induction group | Consolidation group | P | Induction group | Consolidation group | P |
| N | 161 | 27 |  | 162 | 26 |  |
| Age (mean (SD)), years | 63.6 (7.4) | 63.9 (10.3) | 0.863 | 63.2 (7.6) | 59.6 (10.1) | 0.261 |
| Sex (%) |  |  | 1.000 |  |  | 0.165 |
| Male | 133 (82.6） | 22 (81.5） |  | 135 (83.2) | 24 (92.6) |  |
| Female | 28 (17.4) | 5 (18.5) |  | 27 (16.8) | 2 ( 7.4) |  |
| Smoking history (%) |  |  | 0.008 |  |  | 0.425 |
| No | 92 (57.1) | 21 (77.8) |  | 98 (60.5) | 12 (46.6) |  |
| Yes | 69 (42.9) | 6 (22.2) |  | 64 (39.5) | 14 (53.4) |  |
| Drinking history (%) |  |  | 0.029 |  |  | 0.268 |
| No | 94 (58.4) | 18 (66.7) |  | 98 (60.2) | 11 (41.0) |  |
| Yes | 67 (41.6) | 9 (33.3) |  | 64 (39.8) | 15 (59.0) |  |
| ECOG score (%) |  |  | 0.016 |  |  | 0.507 |
| 0-1 | 144 (89.4) | 19 (70.4) |  | 134 (82.7) | 20 (79.7) |  |
| 2 | 17 (10.6) | 8 (29.6) |  | 28 (17.3) | 6 (20.3) |  |
| BMI (mean (SD)) | 23.1 (3.4) | 23.2 (3.1) | 0.793 | 22.9 (3.4) | 20.5 (5.0) | 0.295 |
| Tumor location (%) |  |  | 0.823 |  |  | 0.253 |
| Upper | 73 (45.3) | 14 (51.9) |  | 73 (45.1) | 18 (68.2) |  |
| Middle | 59 (36.6) | 8 (29.6) |  | 56 (34.6) | 3 (12.6) |  |
| Lower | 29 (18.1) | 5 (18.5) |  | 33 (20.3) | 5 (19.2) |  |
| Tumor length (mean SD)), cm | 5.5 (2.9) | 7.3 (4.7) | 0.011 | 5.8 (3.2) | 6.5 (3.1) | 0.246 |
| Clinical T stage (%) |  |  | 0.521 |  |  | 0.438 |
| T1-2 | 55 (34.2) | 8 (29.6) |  | 52 (32.7) | 5 (20.8) |  |
| T3-4 | 106 (65.8) | 19 (70.4) |  | 110 (67.3) | 21 (79.2) |  |
| Clinical N stage (%) |  |  | 0.346 |  |  | 0.562 |
| N0 | 5 (3.1) | 1 (3.7) |  | 5 (3.1) | 1 (3.8) |  |
| N+ | 156 (96.9) | 26 (96.3) |  | 157 (96.9) | 25 (96.2) |  |
| Clinical M stage (%) |  |  | 0.251 |  |  | 0.894 |
| M0 | 139 (86.3) | 21 (77.8) |  | 137 (84.9) | 21 (82.4) |  |
| M1 | 22 (13.7) | 6 (22.2) |  | 25 (15.1) | 5 (17.6) |  |
| Clinical TNM stage (%) |  |  | 0.348 |  |  | 0.597 |
| I-II | 12 (7.4) | 3 (11.2) |  | 11 ( 6.5) | 1 ( 3.8) |  |
| III-IV | 149 (92.5) | 24 (88.8) |  | 151 (93.5) | 25 (96.2) |  |
| ICIs cycle (%) |  |  | 0.041 |  |  | 0.574 |
| 1-2 | 69 (42.9) | 5 (18.5) |  | 64 (39.4) | 14 (55.0) |  |
| 3-4 | 32 (19.9) | 5 (18.5) |  | 32 (19.7) | 2 (7.7) |  |
| 5-6 | 16 (9.9) | 3 (11.1) |  | 15 ( 9.4) | 2 (7.7) |  |
| ≥7 | 44 (27.3) | 14 (51.9) |  | 51 (31.5) | 8 (29.6) |  |

Abbreviation: *BMI* = body mass index, *ECOG* = Eastern Cooerpative Oncology Group, *ICIs =* immune checkpoint inhibitors, *SD* = standard deviation, *sIPTW* = stabilized inverse probability of treatment weighting.

Supplementary table 7: Patient characteristics in concurrent and consolidation groups before and after sIPTW-adjustment.

| Group | Unmatched | | | sIPTW | | |
| --- | --- | --- | --- | --- | --- | --- |
|  | Concurrent group | Consolidation group | P | Concurrent group | Consolidation group | P |
| N | 102 | 27 |  | 101 | 22 |  |
| Age (mean (SD)), years | 64.2 (7.8) | 63.9 (10.3) | 0.894 | 63.5 (7.8) | 64.5 (11.3) | 0.791 |
| Sex (%) |  |  | 1.000 |  |  | 0.482 |
| Male | 81 (79.4） | 22 (81.5） |  | 79 (78.3) | 18 (85.4) |  |
| Female | 21 (20.6) | 5 (18.5) |  | 22 (21.7) | 4 (14.6) |  |
| Smoking history (%) |  |  | 0.002 |  |  | 0.229 |
| No | 45 (44.1) | 21 (77.8) |  | 53 (52.2) | 15 (70.3) |  |
| Yes | 57 (55.9) | 6 (22.2) |  | 48 (47.8) | 7 (29.7) |  |
| Drinking history (%) |  |  | 0.028 |  |  | 0.513 |
| No | 50 (49.0) | 18 (66.7) |  | 56 (55.4) | 13 (59.9) |  |
| Yes | 52 (51.0) | 9 (33.3) |  | 45 (44.6) | 9 (40.1) |  |
| ECOG score (%) |  |  | 0.013 |  |  | 0.782 |
| 0-1 | 90 (88.2) | 19 (70.4) |  | 82 (81.1) | 17 (78.6) |  |
| 2 | 12 (11.8) | 8 (29.6) |  | 19 (18.9) | 5 (21.4) |  |
| BMI (mean (SD)) | 22.9 (3.1) | 23.2 (3.1) | 0.658 | 22.9 (3.0) | 21.9 (3.9) | 0.464 |
| Tumor location (%) |  |  | 0.093 |  |  | 0.475 |
| Upper | 41 (40.2) | 14 (51.9) |  | 44 (43.2) | 13 (61.6) |  |
| Middle | 52 (51.0) | 8 (29.6) |  | 47 (46.6) | 7 (30.3) |  |
| Lower | 9 (8.8) | 5 (18.5) |  | 10 ( 10.2) | 2 ( 9.2) |  |
| Tumor length (mean (SD)), cm | 5.8 (2.2) | 7.3 (4.7) | 0.026 | 6.1 (2.4) | 6.0 (3.9) | 0.934 |
| Clinical T stage (%) |  |  | 0.041 |  |  | 0.512 |
| T1-2 | 54 (52.9) | 8 (29.6) |  | 47 (47.2) | 7 (33.2) |  |
| T3-4 | 48 (47.1) | 19 (70.4) |  | 54 (52.8) | 15 (66.8) |  |
| Clinical N stage (%) |  |  | 0.330 |  |  | 0.137 |
| N0 | 11 (10.8) | 1 (3.7) |  | 9 ( 8.8) | 1 ( 4.5) |  |
| N+ | 91 (89.2) | 26 (96.3) |  | 92 (91.2) | 21 (95.5) |  |
| Clinical M stage (%) |  |  | 0.313 |  |  | 0.985 |
| M0 | 90 (88.2) | 21 (77.8) |  | 86 (85.7) | 18 (85.3) |  |
| M1 | 12 (11.8) | 6 (22.2) |  | 15 (14.3) | 4 (14.7) |  |
| Clinical TNM stage (%) |  |  | 0.380 |  |  | 0.265 |
| I-II | 16 (15.7) | 3 (11.2) |  | 13 (12.6) | 1 (4.5) |  |
| III-IV | 86 (84.3) | 24 (88.8) |  | 88 (87.4) | 21 (95.5) |  |
| ICIs cycle (%) |  |  | 0.724 |  |  | 0.199 |
| 1-2 | 11 (10.8) | 5 (18.5) |  | 13 (13.1) | 6 (25.8) |  |
| 3-4 | 19 (18.6) | 5 (18.5) |  | 18 (17.4) | 2 ( 9.4) |  |
| 5-6 | 12 (11.8) | 3 (11.1) |  | 14 (13.4) | 7 (32.4) |  |
| ≥7 | 60 (58.8) | 14 (51.9) |  | 56 (56.1) | 7 (32.4) |  |

Abbreviation: *BMI* = body mass index, *ECOG* = Eastern Cooerpative Oncology Group, *ICIs =* immune checkpoint inhibitors, *SD* = standard deviation, *sIPTW* = stabilized inverse probability of treatment weighting.
